# Epigenetics in Knee Osteoarthritis: A 2020–2023 Update Systematic Review

**DOI:** 10.3390/life14020269

**Published:** 2024-02-17

**Authors:** Davide Caldo, Eugenia Massarini, Massimiliano Rucci, Silvia Deaglio, Riccardo Ferracini

**Affiliations:** 1 Department of Medical Sciences, University of Torino, 10126 Turin, Italy; 2 Immunogenetics and Transplant Biology Unit, Città della Salute e della Scienza University Hospital, 10126 Turin, Italy; 3 Dipartimento di Scienze Chirurgiche e Diagnostiche Integrate, Università di Genova, 16126 Genua, Italy; 4 Ospedale Koelliker, Corso Galileo Ferraris 247/255, 10134 Turin, Italy

**Keywords:** systematic review, knee osteoarthritis, miRNA, lncRNA, epigenomics, epigenetics, DNA methylation, histone modification, non-coding RNA, micro-RNA, histone methylation, histone acetylation

## Abstract

Osteoarthritis is a leading cause of disability in the world. The scientific literature highlights the critical importance of epigenetic regulatory effects, intertwined with biomechanical and biochemical peculiar conditions within each musculoskeletal district. While the contribution of genetic and epigenetic factors to knee OA is well-recognized, their precise role in disease management remains an area of active research. Such a field is particularly heterogeneous, calling for regular analysis and summarizing of the data that constantly emerge in the scientific literature, often sparse and scant of integration. The aim of this study was to systematically identify and synthesize all new evidence that emerged in human and animal model studies published between 2020 and 2023. This was necessary because, to the best of our knowledge, articles published before 2019 (and partly 2020) had already been included in systematic reviews that allowed to identify the ones concerning the knee joint. The review was carried out in accordance with Preferential Reporting Items for Systematic Reviews and Meta-Analyses (PRISMA) guidelines. Only peer-reviewed articles were considered for inclusion. A total of 40 studies were identified, showing promising results in terms either of biomarker identification, new insight in mechanism of action or potential therapeutic targets for knee OA. DNA methylation, histone modification and ncRNA were all mechanisms involved in epigenetic regulation of the knee. Most recent evidence suggests that epigenetics is a most promising field with the long-term goal of improving understanding and management of knee OA, but a variety of research approaches need greater consolidation.

## 1. Introduction

Osteoarthritis (OA) affects >40% of individuals over the age of 70 years and is a leading cause of pain and loss of physical function [1,2].

The causes of OA are complex and multifactorial, involving genetic and environmental factors [3]. The genetic contribution to OA is well—but not completely—established, with several single nucleotide polymorphisms (SNPs) from several genes that have recently been linked to OA susceptibility [4] and genome-wide association studies that uncovered a likely role in OA for the genes encoding structural extracellular matrix components [5]. Current evidence in the literature available on the subject supports the theory of a polygenic disease, as the studies reported a wide variety of OA-associated loci [6,7,8,9].

Knee OA (KOA), in socioeconomical terms, is a common condition that affects over 300 million people worldwide Typical clinical manifestations include strong and continuous pain, functional impairment, swelling and others, representing a major cause of absence from work, withdrawal from sport activity and other limitations [10].

In eukaryotes, genetic expression is dynamically regulated at the chromatin level by epigenetics, defined as a group of chemical modifications that are both reversible and inheritable changes in gene expression without alterations in the underlying DNA nucleotide sequence; epigenetic mechanisms can be affected by environmental factors such as Body Mass Index (BMI), smoking and inflammation and can be transmitted from one generation of cells to the others [11]; and the molecular basis of epigenetic mechanisms is complex and comprises DNA methylation, modifications of histones and gene expression regulation by non-coding RNAs [11]. The literature has shown that more than 80% of OA-related versions are located in non-coding regions of the human genome [12]. It has then been proposed that, more than changes to the genome sequence itself, changes in gene expression might influence OA development [13]. From this aspect came the necessity of analyzing epigenetics, a fundamental gene–environment interaction and regulation process, as a possible leading cause of OA onset and progression. Epigenetics regulation, being more versatile than standard genomic modifications, includes DNA methylation and other forms of post-transcriptional control [14]. DNA methylation is a biochemical modification of DNA that involves the addition of a methyl group (-CH₃) to a nitrogenous base. In eukaryotes, DNA methylation plays a role in regulating gene expression: genes that are methylated are often silenced, meaning that they are not transcribed into RNA. This can be important for controlling development, cell differentiation and other processes [11]. The structure of DNA is wrapped around histone proteins to form nucleosomes, controlled by histone modifications (e.g., acetylation, methylation, etc.) that can either loosen or tighten their structure, affecting the accessibility of DNA to transcription factors [11]. The regulation of gene expression by histone modifications is a complex and dynamic process, essential for a variety of cellular processes. Finally, non-coding RNA (ncRNA) in molecular biology is a genetic transcript that does not undergo translation, simply resulting in an RNA molecule capable of a variety of functions: it can be ribosomal RNA (rRNA), transfer RNA (tRNA) or it can be a component of enzymatic complexes involved in the processes of transcription, replication, splicing and other processes related to gene expression. Among ncRNAs, long non-coding RNAs (lncRNAs), micro-RNA and circRNA can be cited. These RNAs are the subject of current research and seem to be involved in many biological mechanisms: splicing, miRNA storage, transcriptional and post-transcriptional processes and a range of pathologies associated with them [11].

The discovery of an epigenetic role in OA has essential implications for developing new treatments [15]. Targeting epigenetic changes may slow the progression of the disease and improve patient outcomes. Some examples of environmental factors that may affect OA progression through epigenetic mechanism include diet (studies have shown that a diet high in saturated fat and cholesterol can increase the risk of OA), exercise (generally beneficial for people with OA, but it can also increase inflammation that may lead to epigenetic changes that further damage the joint) [16] and toxins (such as cigarette smoke and heavy metals that can increase the risk of OA) [17]. Early genetic and epigenetic scanning for risk profiles, together with a more precise radiographic discovery of localized chondral focal and diffuse disease, are a key stone for future knee OA early treatment. Current evidence, for example, values the sensitivity of the MRI, the most common and accessible exam for diagnosing OA, somewhere between 45 and 94% [18,19,20].

Other diagnostic tools, such as acoustic signals analysis with or without artificial intelligence involvement, offer promising perspectives for future development [21,22].

Earlier studies explored the role of epigenetic factors involved in OA regardless of the specificity of an articular district; the influence of genetic variation in OA susceptibility varies depending on the joint involved, the load distribution and the unique microenvironment of each joint [23]. It became evident that information focused on epigenetics mechanisms in a specific district is warranted, as well as regular analysis and summary of data constantly emerging in the scientific literature, often sparse and scant of integration. We aimed to identify and synthesize all new evidence that emerged in human and animal model studies published in the years between 2020 and 2023. This was because, to the best of our knowledge, articles up to 2019 (and partly 2020) had already been included in previous systematic reviews that were allowed to identify the ones related to the knee joint [23].

## 2. Methods

### 2.1. Review Design

A review of the literature was carried out following the Preferential Reporting Items for Systematic Reviews and Meta-Analyses (PRISMA) guidelines.

The systematic research included studies describing the influence of different epigenetic factors on the development or progression of KOA. The question investigated was “How are epigenetic factors contributing to the development of KOA and what are the mechanisms involved?”.

The inclusion criteria were in vitro or in vivo models of KOA based on knee-derived cells and tissues from patients of any age with clinically diagnosed KOA or in vitro and animal models of knee osteoarthritis, English-written papers and publication dates between January 2020 and October 2023.

**Figure 1:**
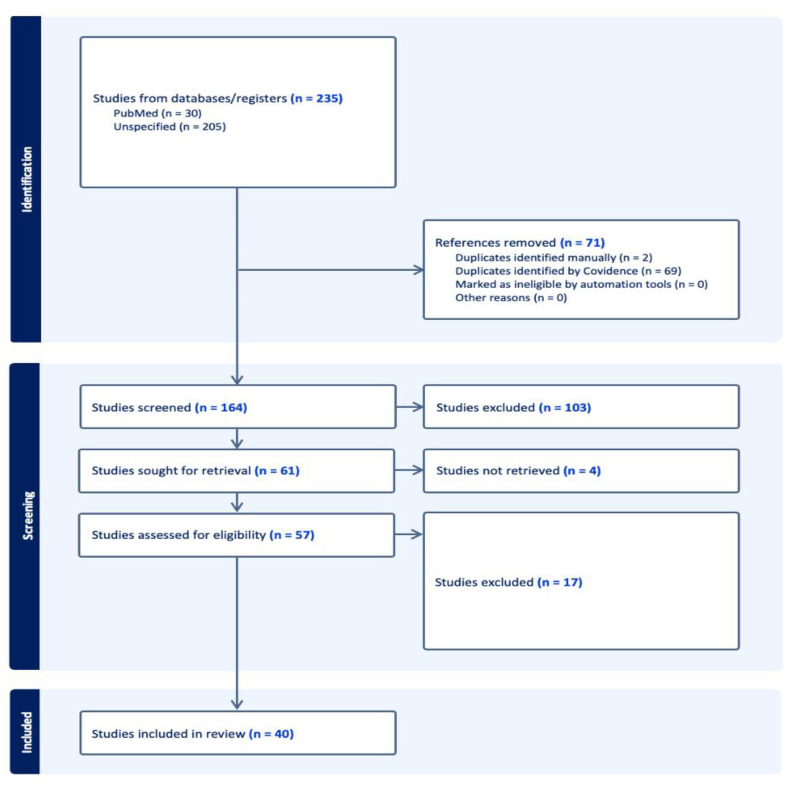
PRISMA flowchart illustrating the workflow diagram of the study selection process, according to the PRISMA guidelines.

Articles on OA localized in joints other than the knee and studies involving genetic mechanisms (i.e., gene polymorphism) or environmental factors with uninvestigated epigenetic features were excluded. The complete workflow is available as a block diagram in Figure 1.

### 2.2. Search Strategy

Keywords were identified exploiting the MeSH (Medical Subject Headings), the NLM-controlled thesaurus used for indexing articles for PubMed. The keywords used to conduct the research in databases were knee osteoarthritis AND epigenetics, epigenomics, methylation, histone modification, noncoding RNA, microRNA, circRNA, long noncoding RNA, smallRNAs, and histone acetylation. Keywords, for example, were combined as follows: “knee osteoarthritis AND epigenetics, OR epigenomics, AND noncodingRNA, OR microRNA”. A list of all combinations and their relative output is available in the Section 3.

### 2.3. Study Selection

Authors mined all published studies available in PubMed by October 2023. A further manual research into the bibliography of selected articles was conducted parallel to reduce potential omission of additional appropriate documents.

A thorough screening of titles and abstracts by two reviewers (DC and EM) resulted in reviewing selected full-text articles, according to the PRISMA flowchart; author exploited the guidance of Covidence app.

Three reviewers (DC, MR and EM) screened the full articles and reached a consensus for final inclusion, then extracted the data. Consensus on the possible inclusion, or exclusion, of the studies found was reached through a vote in the panel of three researchers. Studies with a minimum of two positive votes were collected for data extraction. The extracted data concerning the study design, number of patients, demographics of patients, pathology definition, biological sample, gene/s involved, type of analysis and results are summarized in Table 1 (in vitro and animal models) and Table 2 (human).

## 3. Results

The keywords used to conduct the research in databases retrieved the following results: knee osteoarthritis AND epigenetics (76 articles), knee osteoarthritis AND epigenomics (13 articles), knee osteoarthritis AND methylation (104 articles), knee osteoarthritis AND histone modification (6 articles), knee osteoarthritis AND noncoding RNA (328 articles), knee osteoarthritis AND microRNA (267 articles), knee osteoarthritis AND circRNA (24 articles), knee osteoarthritis AND long noncodingRNA (73 articles) and knee osteoarthritis AND histone acetylation (6 articles).

**Figure 2:**
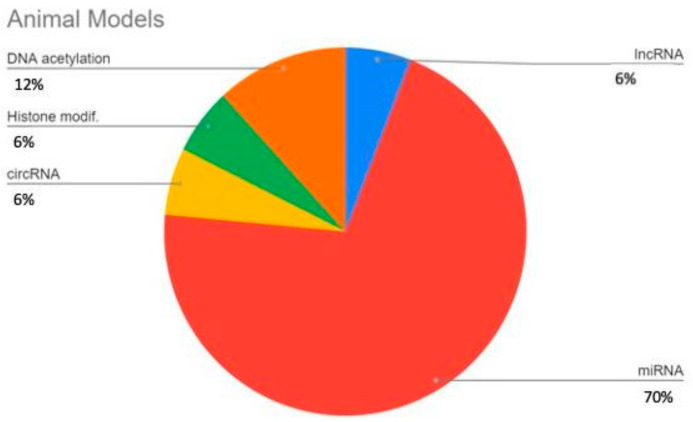

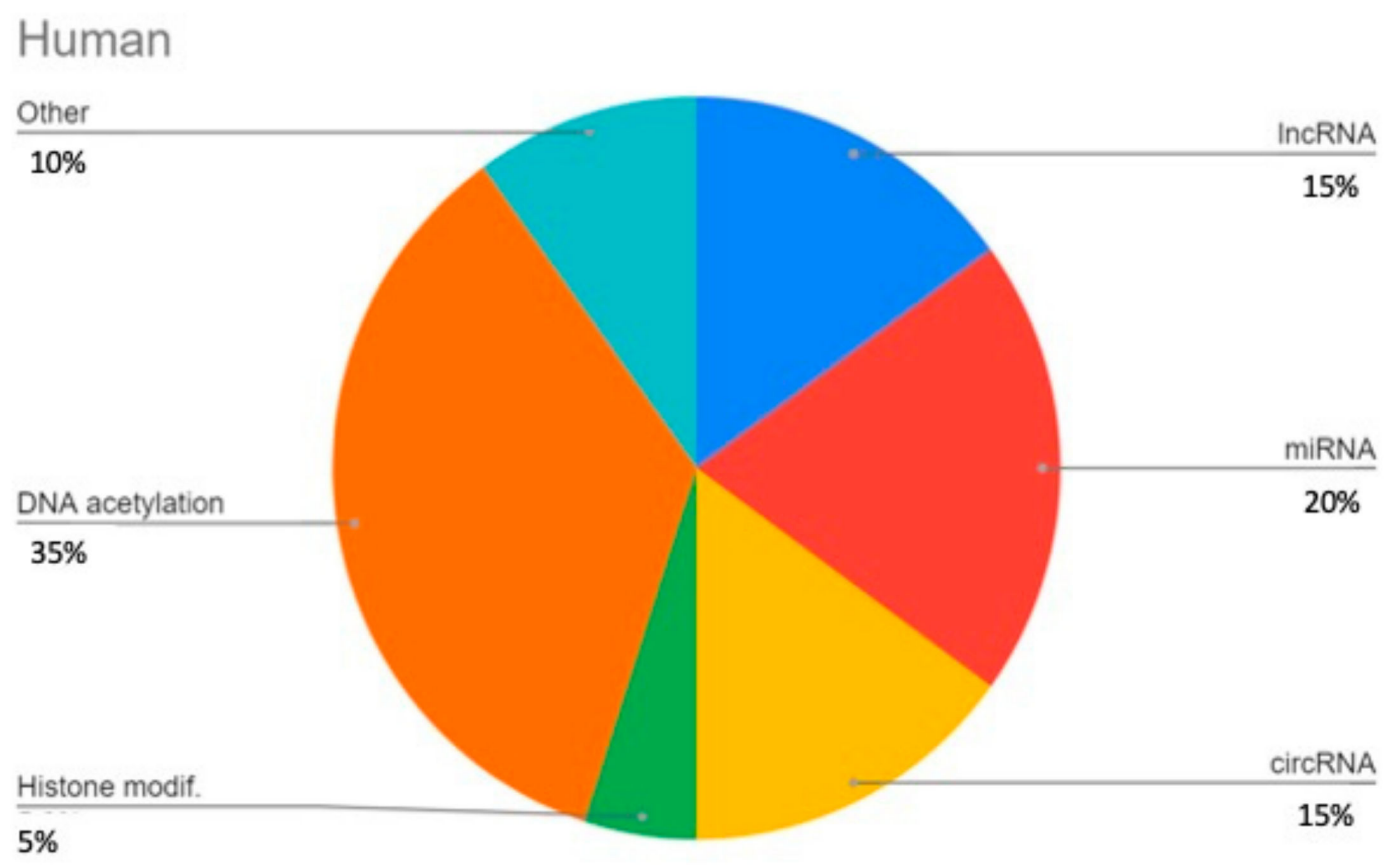
Percentage distribution of studies concerning each epigenetic regulatory agent both in animal models (**image above**) and human ex vivo studies (**image below**)—some studies investigated more than one possible mechanism of regulation; studies based on animal models were 19 in total, while studies based on retrieved human tissues were 21.

**Figure 3:**
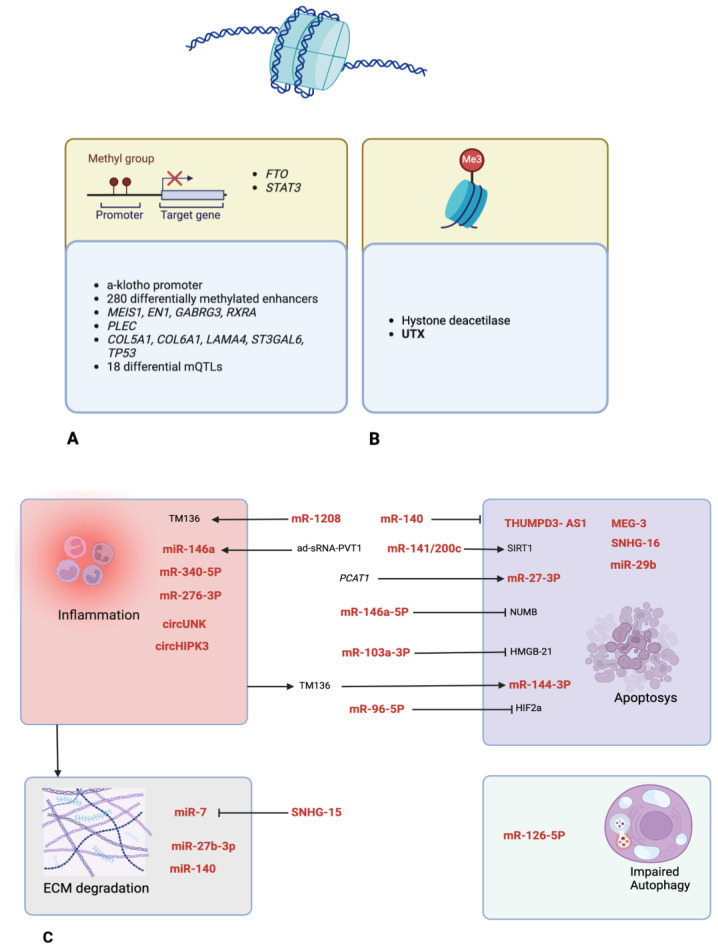
Epigenetic regulation molecular agents. (**A**) Genes responsible for differential methylation (upper box) and regulated target genes/regions (lower box). (**B**) Histone modification enzymes. (**C**) Main ncRNAs and their relationship to 4 mechanisms involved in KOA pathogenesis: inflammation, cell apoptosis, ECM degradation and impaired autophagy; arrows indicate induction while T-ends indicate inhibition effects; and ncRNAs in red, other molecules and genes are depicted in black. All images were created with BioRender.com.

All references retrieved with all possible combinations of keywords were imported into the Covidence app; after discarding duplicates, 164 journal articles were included in the study. Based on the title and abstract scanning and consensus between two blinded reviewers (DC and EM), 103 studies were excluded due to inappropriateness (studies concerning gene mutations, gene polymorphism, etc.), wrong outcome (rheumatoid arthritis) or study type (review). In the second round of review based on full texts, 40 articles were selected for assessment. The distribution of investigated epigenetic mechanisms described throughout the articles is illustrated in Figure 2. All molecules investigated in papers are summerized in Figure 3.

### 3.1. LncRNAs

LncRNAs are transcripts longer than 200 nt and thought to be non-functional in the past. They can act as “sponges” for one or more miRNAs to silence their expression. miRNAs are small (20 to 24 nt) single-stranded, non-coding RNAs that can have several roles in post-transcriptional regulation of gene expression, as they can inhibit mRNA translation. They can be abnormally expressed in KOA tissues and become key factors for KOA development (as shown in Table 1 and Table 2).

The publications included in this systematic review demonstrate that lncRNA PVT1 plays a role in cartilage degeneration and is associated with the severity of the disease. The effectiveness of ad-siRNA-PVT1 in controlling joint inflammation in diabetic OA mice is linked to the suppression of miR-146a expression, pro-inflammatory cytokines and activation of the TGF-β/SMAD4 pathway [24].

In a study by Chen et al., SNHG-15, an lncRNA, was found to be hypermethylated in KOA chondrocytes, leading to its lower effect. SNHG-15 artificial over-expression, on the other hand, inhibited extracellular matrix (ECM) degradation and promoted chondrocyte formation, both in vivo and in vitro. SNHG-15 promotes the KLF4 pathway and inhibits b-catenin by sponging miR-7 (miR-7, on the other hand, directly suppresses KLF4) [25].

In a very interesting study, van Hoolwerff and collaborators [26] identified robustly differentially expressed lncRNAs in KOA, provided insight into their regulatory networks and demonstrated that both intergenic and antisense lncRNAs play an equally important role in the pathophysiology of KOA.

Fan H et al. [27] found that lncRNA *SNHG16* is up-regulated in KOA tissues compared to normal joint tissue, while *SNHG16* knockdown significantly decreases apoptosis by down-regulating the expression level of cleaved caspase-3. Additionally, one study [28] highlighted the pro-apoptotic role of lncRNA *PCAT-1* through expression of miR-27-3p. Finally, the study by Wang Y et al. [29] revealed that over-expression of lncRNA *THUMPD3-AS1* can alleviate cell apoptosis and facilitate inflammatory responses by increasing the expression levels of IL-6, TNF-α and NO via phosphorylation of target proteins NF-κB p65 and MAPk p38.

Li X et al. [30] found a potential correlation between lncRNA CYTOR and gene NRP1, since it demonstrated how NRP1 co-expressed with CYTOR, both up-regulated in KOA cartilage. This correlation might depend on NRP1 capacity to induce vascularization and angiogenesis, both contributing to structural damage and pain.

Wang and colleagues explored the involvement of both lncRNAs and miRNAs in the occurrence and development of KOA; they found that the expression of 186 lncRNAs and 684 miRNAs were differentially expressed in the model of chondrocyte degeneration, providing new insights into the molecular mechanisms of chondrocyte degeneration in KOA and providing potential targets for the differential diagnosis and therapy of OA [31].

### 3.2. miRNAs

miRNAs are small non-coding RNAs, with an average 22 nucleotides in length; such small highly conserved non-coding RNA molecules sustain the regulation of gene expression.

The role of miR-146a-5p was also analyzed in an interesting study [32], where the authors found a relationship with a protein named NUMB, involved in chondrocyte apoptosis and autophagy processes through the Wnt-b-catenin pathway: miR-146a-5p promotes apoptosis and reduces autophagy in chondrocytes. miR-146a-5p was found to be highly expressed in KOA tissue, while NUMB was down-regulated by miR-146a-5p. miR-146a-5p antagonist intra-articular injection positively affected the KOA progression.

Recent studies have shown that miRNAs also play an important role in mitophagy, a process of autophagy that degrades damaged or dysfunctional mitochondria. In particular, miR-126-5p has been found to inhibit autophagy in cartilage cells, acting as an upstream negative regulator for PGC1a. PGC1a is a regulator of mitophagy and biogenesis pathway, decreased in quantity in KOA. Inhibition of miR-126-5p led to increased mitophagy and mitochondrial degradation, resulting in improved resistance to cartilage injury. Mitochondria are powerful regulators of chondral homeostasis, and their disruption was found to be associated with KOA [33].

In another manuscript, miR-103a-3p was down-regulated in KOA tissue and in chondrocytes treated in-vitro with LPS. Over-expression of miR-103a-3p inhibits chondrocyte apoptosis and inflammation in OA, which appears to be an attractive approach to OA treatment. This effect was attributed to the negative regulation exerted by miR-103a-3p on HMGB1, an important positive mediator of inflammation [34].

Another study from Endisha et al. found that miR-34a-5p expression is increased in the cellular environment of patients with KOA (cartilage and synovium). It was also increased in the knees of mice fed with a high-fat diet. Intra-articular injection of miR-34a-5p had a pro-inflammatory effect with the production of core markers for the KOA disease. The synovium and cartilage of mice treated with intra-articular injections of miR-34a-5p showed signs of OA-like degeneration (cartilage fissuring, increased OA scores, synovial thickening, etc.). Injection of the antisense oligonucleotide protected the joint from the effects of degeneration, even though not all typical inflammation markers were affected [35].

An increased level of miR-27b-3p was also observed in the synovial fluid of patients with late-stage KOA in a study by Tavallaee et al. The levels of this miRNA appeared to be associated with OA severity score. Effects of injection of miR-27b-3p mimic in knees of an animal murine model were summarized in a synovial fibrosis-like phenotype, increased synovitis scores and inflammation marker production, plus a pro-fibrotic response in fibroblast-like synoviocytes [36].

According to the results presented in a manuscript by Yi et al., *ONECUT2* and *SMURF2* genes were significantly up-regulated in the KOA group and in the FSCs (Fibroblast Synovial Cells) stimulated by IL-1b in vitro. IL-1b induced the expression of *ONECUT2*, *SMURF2* and other markers of inflammation. Bioinformatic analysis revealed that *ONECUT2* was the binding site of miR-144-3p. This miRNA was therefore down-regulated by IL-1b. miR-144-3p was found to be the target of lncRNA TM1-3p, which is up-regulated by IL-1b in FSCs. Over-expression of TM1-3p promoted cell viability and proliferation while inhibiting apoptosis; results were reversed with a high level of miR-144-3p. Inhibition of TM1-3p was proposed as a putative therapy for KOA in mice [37].

A strongly increased level of SDC-4, a membranous receptor member of the syndecans family, was identified in OA cells. This is a key component implicated in the matrix degradation process. Hypoxia can influence production of SDC-4 mRNA and protein. HIF-2a (Hipoxia-Induced Factor 2a) is produced in low-oxygen conditions and is involved in cartilage degradation. Both SDC-4 and HIF-2a participate in KOA pathogenesis. miR-96-5p was found to be up-regulated post-SDC-4 inhibition. Such up-regulation inhibited HIF-2a activity too. Anti-SDC-4 inhibited HIF-2a expression through miR-96-5p up-regulation, attenuated cartilage degeneration in OA mice and inhibited a long list of markers of inflammation [38].

HucMSCs–EVs (Human Umbilical Cord Mesenchymal Stem Cells-Extracellular Vesicles), secreted by cells as an intercellular communication method and filled with different types of mediators, slowed down the progression of KOA, according to the results of a study by Zhou et al., increasing protective factor production and decreasing inflammation. HucMSCs-EVs, identified through surface antigens CD90 and CD105, and the miRNAs contained in the sEVs could decrease the m6a level of *NLRP3* mRNA in macrophages via miR-1208 binding to METTL3 and thus inactivating it. (*METTL3* is the key component of the m6a mRNA methyltransferase in eukaryotic cells.) NLRP3 inflammasome activates caspase-1, which cleaves pro-inflammatory factors. HucMSCs-EVs lose their protective effect in KO mice for NLRP3 (NLRP3^−/−^) [39].

Another manuscript by Jin et al. evaluated the effect of exosomes from human bone marrow MSCs (Mesenchymal Stem Cells) in treating KOA. A key component, EVs, and the miRNAs contained in them were studied for efficacy. Both BM-MSCs and BM-MSC-derived exosomes alleviated cartilage destruction and subchondral bone remodeling in a KOA rat model. In vitro, assays showed that BM-MSCs exosomes could maintain the chondrocyte phenotype (increased collagen type II synthesis and inhibited IL-1β-induced senescence and apoptosis). Exosome lncRNA MEG-3 also reduced the senescence and apoptosis of chondrocytes induced by IL-1β, indicating that lncRNA MEG-3 might partially account for the anti-OA effects of BM-MSCs exosomes [40].

One manuscript by Ji et al. evaluated the therapeutic efficacy of chondrocyte-specific aptamer-decorated tgg2-PEGylated polyamidoamine (PAMAM) nanoparticles (NPs)-based miRNAs delivery system for KOA treatment. NPs were designed specifically for the purpose, and their efficacy was positively evaluated. miR-141/200c was then studied as a possible central actor in OA development, being up-regulated in the process, affecting proliferation, apoptosis and cell growth by targeting SIRT1, which activates the IL-6/STAT3 pathway. Cartilage-specific silencing of miR-141/200c, perhaps via an miR-141/200c inhibitor contained in NPs, could reverse chondral degeneration [41].

In a manuscript by He et al., the combination of drug therapy (lornoxicam, a COX-2 inhibitor) and gene therapy (miR-140) was tested for efficacy in cationic liposomes as therapy carriers. These carriers can enter the cell by fusion with the outer membrane or by endocytosis. The combination of the two treatments together resulted in more effectiveness than the two, if tested alone; miR-140 is strongly expressed in normal cartilage, much less in OA cartilage. Its inhibition induces OA-like changes in vivo and, when up-regulated, it can regulate gene expression in chondrocytes in vitro and moderate KOA in vivo in a mice model [42].

A significant increase in the number of senescent fibroblast-like synoviocytes (FLSs) was observed in patients and mouse models with progressing OA. These senescent FLSs exhibited impaired autophagy, primarily attributed to a reduced expression of an essential enzyme involved in autophagosome formation. This down-regulation was found to be mediated by excessive methylation of the enzyme’s RNA, leading to its instability and enhanced production of the senescence-associated secretory phenotype (SASP). The crucial role of FLS senescence in the progression of KOA was highlighted, and a therapeutic target involving siRNA-mediated enzyme silencing was proposed [43].

Intra-articular injection of infrapatellar fat pad–Mesenchymal Stem Cells (IFP–MSCs) had a strong immunomodulatory effect through their CD10-bound small extracellular vesicles (IFP–MSC sEVs). Synovium and infrapatellar fat pad (IFP) can be the origins of pro-inflammatory molecules in KOA. The different therapeutic potential of CD10-High and CD10-Low sEVs was tested in vivo and in vitro (animal model of synovial inflammation) in a manuscript by Kouroupis et al.: CD10-High and -Low vesicles were found to have different miRNA profiles. CD10-High sEVs can have a significant chondroprotective role by inactivating SubstanceP, a pro-inflammatory and pro-fibrosis IFP marker, via CD10 itself [44].

One study (previously mentioned in the “animal model” above) had a significant section dedicated to test human samples. In such study, Talavee and colleagues [36] found increased micro-RNA-27b-3p (miR-27b-3p) levels in synovial fluid of patients with late-stage radiographic KOA, given its role in modulating synovial ECM regulatory networks involved in synovial fibrosis.

Similarly, ECM degradation occurring in KOA was shown to be driven by the expression of MMP13 and ADAMTS5, which are primary cartilage matrix-degrading enzymes, recognized to be targeted by miR-140 [45].

Finally, Dou P et al. [46] found that DNM3TB could methylate miR-29b, which is involved in the regulation of chondrocyte proliferation and apoptosis by targeting PTLH expression and thus enhancing CDK4. In turn, CDK4-induced ubiquitination of RUNX2 led to apoptosis suppression.

### 3.3. circRNA

Circular RNA (circRNA), a distinct type of single-stranded RNA, differs from conventional linear RNA by its unique covalently closed-loop structure. Unlike linear RNA, where the 3′ and 5′ ends are free, circRNAs exhibit a seamless connection between these termini, forming a continuous circle. This distinctive feature imparts a remarkable array of properties to circRNAs, many of which have only recently been elucidated, pertaining epigenetic control of gene expression [11].

Aerobic exercise and glucosamine hydrochloride capsules (OTL), glucosamine being the most important component of cartilage tissue, both have a therapeutic effect on KOA, if used alone. One study by Fang et al., therefore, investigated the combined effect of these two treatments: they were found to be synergistic if used together in their positive effect on OA progression (reduced quantity of inflammatory markers and better histologic scores). At the same time, an over-expression of circUNK, a circular RNA (circRNA) related to KOA, was found to be protective for MIA-induced OA-like chondrocyte inflammation [47].

Liu P et al. [48] identified a total of 122 differentially expressed circRNAs, among which 89 were down-regulated and 33 were up-regulated. Hsa_circ_0072697 may play an important role in the pathogenesis of KOA.

Similarly, another study [49] revealed differential expression of circRNAs in patients affected by KOA and Kashin–Beck disease. Hsa_circRNA_0020014 was shown to be the most significantly differentially expressed molecule between OA and KBD, supporting its potential exploitability in differential diagnosis.

Wu Q et al. [50] demonstrated how CircHIPK3 is significantly up-regulated in KOA cartilage tissue and cells and how it is involved in apoptosis regulation through a negative correlation with its target gene miRNA 124 and downstream target molecule SOX8.

### 3.4. DNA Methylation

The biological process of DNA methylation involves the addition of methyl groups to the DNA molecule, catalyzed by a family of DNA methyltransferases (DNMTs) that transfer a methyl group from S-adenyl methionine to the fifth carbon of a cytosine residue to form 5 mC. When located in the promoter region of a gene, DNA methylation generally suppresses gene transcription [11].

In a study by Cai et al. the role of *FTO* (fat mass and obesity-associated gene, a specific demethylase) in KOA progression was evaluated. *FTO* was diminished in chondrocyte cells with LPS-induced OA. LPS inhibited cell activity and promoted apoptosis and inflammation in a concentration-dependent manner. Over-expressed *FTO* gene, instead, had a positive effect on KOA, through the direct modulation of miR-515-5p. This miRNA, on the other hand, inhibited MyD88/NF-kB pathway in a TLR4-dependent (Toll-Like Receptor 4) mechanism [51].

Another study by Iijima et al. evaluated the effect of ECM stiffening on cartilage aging and on KOA. Increased stiffness in the chondral ECM was discovered to cause the promoter of a-klotho, a longevity protein, to be hypermethylated, leading to its inactivation. This process led to accelerated chondrocyte senescence and cartilage degeneration. It was, in fact, reversible if the same chondrocytes were exposed to a softer ECM. The pathways involved in cartilage degeneration, such as PI3K/AKT, were also found to be enriched and more active in KOA male mice rather than female (heterozygous mice for a-klotho showed KOA progression only in male animals). A-klotho is a key negative regulator of the PI3K/AKT pathway [52].

Lin X et al. [53] investigated the enhancer methylation landscape in KOA patients, and 280 differentially methylated enhancer CpGs were identified in KOA patients. The finding was confirmed in another study [54], which identified 12 OA discriminative methylation sites involved in regulation of genes such as *MEIS1*, *EN1*, *GABRG3* and *RXRA*.

In addition, Sarkar A et al. [55] performed DNA methylation profiling to explore the association between CpG methylation and human chondrocyte age. Researchers found that *STAT3* has a fundamental role in regulating DNA methylation in cartilage disease.

Moreover, KOA association signal was demonstrated to be marked by SNP rs11780978 correlated with methylation of CpGs within *PLEC* [56]. Knocking down plectin showed an increase in expression of genes involved in pathways of the innate and the acquired immune response within joint tissues. Zhang Q et al. [57] contributed to these findings by demonstrating that the elevated production of TNF-a in subjects with OA can be attributed to the suppressed methylation of TNF-a promoter region and that changes in epigenetic status regulate TNF-a expression in the cells, which are pivotal to the OA disease process.

In support of these findings, Jin L et al. [58] analyzed DNA methylation array data to confirm other core genes related to OA. Finally, COL5A1, COL6A1, LAMA4, ST3GAL6 and TP53 were found to be the genes most expressed. Lastly, another study [59] performed a genome-wide methylation profiles analysis to better highlight OA etiopathogenesis and revealed substantial differences between tissue types, underlying the cell type, and OA-grade specificity of DNA methylation. In fact, 18 differential mQTLs between low-grade and high-grade OA cartilage were identified.

### 3.5. Histone Modifications

Histone alterations, such as phosphorylation, methylation, acetylation and more, altogether with their opposite alteration (e.g., demethylation), collectively termed histone modifications, are intricate epigenetic alterations that exert a profound influence on gene expression by modulating chromatin structure. These modifications involve the addition or removal of methyl groups to specific histone residues, thereby regulating the accessibility of DNA to transcription factors and ultimately influencing the expression of genes.

The increased activation and expression of histone deacetylases (HDACs) might be involved in KOA pathogenesis. A study by Wen et al. investigated the effect of intra-articular injection of Panobinostat, an HDAC inhibitor, as a chondroprotective treatment in mice. In this study, weight-bearing capacity on the injured limb and mechanical allodynia was evaluated. The study suggests that histone deacetylases (HDACs) may exert distinct regulatory effects on the chondrocyte phenotype during the initial phases of OA development. These findings offer compelling evidence that Panobinostat holds promise as a potential therapeutic agent for OA. The increased expression of HDACs also modulated miR-146a, a negative controller of inflammation response in KOA [60].

UTX is a histone demethylase that targets di- and tri-methylated histone H3 lysine 27. Lian WS et al. [61] studied how UTX altered the articular cartilage phenotype or KOA development and how it affected H3K27 tri-methylation and epigenomic landscapes, which impacted chondrocyte activity. The interaction between UTX and PRC2 core complex components was shown. In bivalent loci, PRC2 activity acts in concert with UTX to either stimulate cartilage anabolism or activate phenotypic de-differentiation and ECM deterioration through activating Igf2 signaling, thus supporting the evidence that alterations in UTX activity appears to hold a lot of potential with regards to OA.

### 3.6. Others

Duran Sotuela et al. [62] explored how mitochondrial dysfunction and certain mitochondrial polymorphisms play a role in different KOA features. More specifically, the study demonstrated that mtDNA variant m.16519C is over-represented in rapid KOA progressors. Cybrids with this variant show increased mtDNA copy number and decreased mitochondrial biosynthesis, and this is reflected in a higher amount of ROS, less resistance to oxidative stress and impairment of autophagic flux.

Gene expression pattern and its possible implication in KOA therapy has been explored. Park H et al. [63] demonstrated a positive correlation between p16INK4a accumulation in senescent chondrocytes and tissue degeneration, mainly induced by continuous chronic inflammation and extracellular matrix remodeling. On the contrary, the application of p16INK4a siRNA-loaded nanoparticles (p16 si_NPs) specifically inhibited p16INK4a and down-regulated the expression of inflammatory cytokines and MMP13.

**Table 1:** Comprehensive list of retrieved studies on epigenetics modifications leading to KOA development (2020–2023) based on animal models.

Author–Year	Epigenetic Mechanism	Study Design	Main Findings
Wen ZH et al., 2021 [60]	histone modification	Murine animal model of KOA in ACLT mice (4 groups).	The effect of I-A-injected Panobinostat was carried out through MMPs (such as MMP13) activity reduction and less chondral degradation and bone remodeling. This effect was mainly produced via activity modulation of HDAC4 (up-regulated), HDAC6 and HDAC7 (down-regulated) and RUNX2/MMP13 (down-regulated). It also modulated miR-146a, a negative controller of inflammation response in KOA FLSs.
Iijima H et al., 2023 [52]	DNA methylation	4–24-month-old mice (male and female): these ages correspond to ages 20–69 in humans; human articular cartilage tissue from healthy donors.	Increased ECM stiffness drove a-klotho promoter hypermethylation and down-regulated klotho gene expression, accelerating chondrocyte senescence. The inactivation of the a-klotho promoter was attributed to methylation by DNMT1. Exposing the cells to a softer matrix reversed this effect.
Cai D et al., 2023 [51]	DNA methylation	KOA cellular model in chondrocytes treated with LPS; KOA in vivo model in rats via MIA I-A injection.	FTO alleviated KKOA (reduces apoptosis, down-regulates IL-6, IL-1β and TNF-α levels and diminishes COX-2 and iNOS expressions) in an m6a-dependent manner via the miR-515-5p/TLR4/MyD88/NF-κB axis.
Chen X et al., 2022 [43]	ncRNA	Synovial tissue samples from OA and non-OA pts; animal OA model in DMM mice vs. controls; specific cell culture.	Impaired autophagy occurred in OA FLS, accelerating senescence. Increased m6a modifications were observed, together with up-regulated expression of METTL3. METTL3 plays a key role as autophagy suppressor through m6a modification of ATG7, an autophagy-related mRNA in a YTHDF1-dependent manner.
Chen Y et al., 2020 [25]	ncRNA	OA cartilage tissues from 30 patients undergoing TKA vs. 15 normal tissues from amputees; chondrocyte culture; animal murine model (n = 10 OA vs. n = 30 sham) via DMM surgery.	In a KOA murine model, an under-expression of KLF4 and SNHG-15 was detected, with a parallel over-expression of miR-7. SNHG-15 was found to be hypermethylated in OA chondrocytes, leading to its lower effect. SNHG-15 artificial over-expression, on the other hand, inhibited ECM degradation and promoted chondrocyte formation.
Cheng M et al., 2020 [34]	ncRNA	Animal model: 12 OA rats (via ani I-A injection) vs. 12 controls; in vitro model: chondrocyte cell culture treated with LPS (4 groups of treatment).	miR-103a-3p is down-regulated in tissues from OA rats and LPS-treated chondrocytes. Up-regulation of miR-103a-3p promotes cell proliferation and reduces inflammation and apoptosis. HMGB1 (OA-related) is a target of miR-103a-3p. Knockdown of HMGB1 mimics the effect of miR-103a-3p.
Endisha H et al., 2021 [35]	ncRNA	Joint tissues and plasma from human OA patients and healthy controls; cell culture for human chondrocytes and FLSs; animal OA model in mice via DMM.	Expression of miR-34a-5p was significantly increased in the plasma, cartilage and synovium of patients with late-stage OA and in the cartilage and synovium of mice subjected to DMM. Expression increased in obese patients with OA. miR-34a mimics increased key OA markers. miR-34a KO and injection of the ASO version caused less cartilage degradation in OA development.
Fang L et al., 2021 [47]	ncRNA	Cultured chondrocytes OA model; in vivo model in rabbits injected with papain.	Aerobic exercise or OTL treatment—both applied alone—relieved the damage of KOA (cartilage tissue lesions, Mankin score, inflammatory cytokine content, etc.). Combined application showed even better effect than the sum of the two taken alone. Transfection of circUNK attenuated MIA-induced effect on cell viability and apoptosis.
Ji M-L et al., 2021 [41]	ncRNA	OA murine model via DMM vs. controls, wt and miR-141/200c KO.	NPs were designed specifically for the purpose of delivering miRNAs and protecting them for a longer period, and their efficacy was positively evaluated. miR-141/200c was then identified and studied as a central actor in KOA development, being up-regulated during OA progression and affecting proliferation, apoptosis and cell growth by targeting SIRT1, which activates IL-6/STAT3 pathway.
Jin Y et al., 2021 [40]	ncRNA	Animal OA model in mice via DMM + ACLT; cultured human chondrocytes.	BM-MSCs-derived exosomes alleviated cartilage destruction and subchondral bone remodeling in the animal model. In vitro, assays showed that BM-MSCs exosomes could maintain the chondrocyte phenotype. Exosome lncRNA MEG-3 also reduced the senescence and apoptosis of chondrocytes and might partially account for the anti-OA effects of BM-MSC exosomes.
Kim D et al., 2021 [33]	ncRNA	Synovial tissue samples from KOA divided in healthy vs. damaged chondral samples; animal KOA model in DMM mice vs. controls; mice cell culture: KOA cases vs. controls.	Loss of PGC1α in chondrocytes due to up-regulation of miR-126-5p during KOA resulted in activation of PRKN-independent mitophagy pathway. Knockdown of PGC1a activated parkin RBR-E3 ubiquitin protein ligase mitochondria autophagy pathway (up-regulation of BCL2 and BNIP3). miR-126-5p is an upstream regulator for PGC1a.
Kouroupis D et al., 2023 [44]	ncRNA	IFP–MSC cultures; animal model of fat pad fibrosis/synovitis via MIA injection.	Synoviocytes exposed to sEVs demonstrated reduced proliferation and altered inflammation-related molecular profiles. CD-10-High sEVs treatment indicated chondroprotective effect by inactivating SubstanceP, a pro-inflammatory and pro-fibrosis IFP marker, via CD10 itself.
Tavallaee G et al., 2022 [36]	ncRNA	Tissue samples from KOA patients and controls; animal murine KOA model via DMM surgery; FLSs cell culture.	An increased level of miR-27b-3p was observed in synovial fluid and synovia of patients with late-stage KOA. I-A injections in knees of an miR-27b-3p mimic produced in a synovial fibrosis-like phenotype increased inflammation marker production, plus a pro-fibrotic response for FLS. This same injection did not appear to influence any change in the cartilage tissue instead. The axis used for signaling in the synovia was the PPARG/ADAMTS8 in FLSs.
Wang YZ et al., 2021 [24]	ncRNA	Animal KOA mice model with diabetes (ACLT, Streptozocin) of 100 mice: 20 controls, 80 KOA cases (20 non-DM, 40 DM, 20 DM treated with pioglitazone).	PVT1 was found to be up-regulated in KOA chondrocytes. PVT1 levels are even higher and miR-146a levels lower in DM KOA knees than in non-DM KOA knees. PVT1 over-expression promoted the apoptosis of chondrocytes and down-regulated miR-146a effect in KOA DM mice. miR-146a-deficient mice can develop early KOA, as it produced a protective effect against KOA. PVT1 activated TGFb/SMAD signal. KOA was found to be histologically worse, too, in KOA diabetic mice.
Yi Y et al., 2022 [37]	ncRNA	Cultured FSCs IL-1b treated to simulate KOA (4 groups); animal KOA model in DMM mice (4 groups).	ONECUT2 and SMURF2 genes were found to be significantly up-regulated in the KOA group, and in the FSCs, stimulated with IL-1b in vitro. ONECUT2 was the binding site of miR-144-3p. This miRNA was therefore down-regulated by Il-1b. miR-144-3p was found to be the target of TM1-3p, that is up-regulated by IL-1b in FSCs. Over-expression of TM1-3p acted as a promotion for cell vitality and proliferation, while inhibiting apoptosis, results reversed with a high level of miR-144-3p.
Zhang H et al., 2021 [32]	ncRNA	22 KOA tissue samples vs. 22 healthy controls; animal KOA model in mice through ACLT (4 groups: sham/ACLT/ACLT + NC (negative control for miR-146)/ACLT + AntagomiR); cell culture.	miR-146a-5p was found to be highly expressed in KOA tissue, while NUMB was down-produced and negatively regulated by miR-146a-5p. miR-146a-5p AntagomiR, injected intra-articularly, had a positive effect on KOA progression.
Zhou H et al., 2022 [39]	ncRNA	KOA model in mice by DMM + ACLT surgery; human chondrocytes cultured from tissue of patients undergoing TKA.	hucMSCs-EVs slowed down the progression of KOA, promoting chondrocyte activity and inhibiting apoptosis. HucMSCs-EVs could decrease the m6a level of NLRP3 mRNA in macrophages, via miR-1208 binding to METTL3 and thus inactivating it. NLRP3 inflammasome-activated caspase-1, all pro-inflammatory factors. HucMSCs-EVs lost their protective effect in KO mice for NLRP3 (NLRP3^−/−^).
Zhou K et al., 2021 [38]	ncRNA	Animal model in ACLT + DMM mice; murine chondrocytes isolation.	A strongly increased level of SDC-4 was identified in KOA cells. This is a key component implicated in matrix degradation process. Hypoxia can influence production of SDC-4 mRNA and protein. HIF-2a (Hipoxia-Induced Factor 2a) is produced as well in low-oxygen situations and is involved in cartilage degradation. Both SDC-4 and HIF-2a participate in KOA pathogenesis. miR-96-5p was found to be up-regulated post-SDC-4 inhibition. Its up-regulation inhibited HIF-2a activity too.
He K et al., 2021 [42]	ncRNA	Rabbit chondrocytes cells cultured; animal KOA model in mice by papain I-A injection in knees.	miR-140 is strongly expressed in normal cartilage, much less in KOA one. Its inhibition induces KOA-like changes in vivo. When up-regulated, it can moderate knee KOA in vivo in mice. A combination of drug therapy (lornoxicam) and gene therapy (miR-140) was then tested for efficacy in cationic liposomes as therapy carrier. The combination of the two treatments together resulted in more effectiveness than the two previous tested alone.

**Table 2:** Comprehensive list of retrieved studies on epigenetics modifications leading to KOA development (2020–2023) based on human tissue samples (ex vivo).

Author–Year	Epigenetic Mechanism	Study Design	Main Finding
Lian WS et al., 2022 [61]	hystone modification	RT-PCR, assessment of OA histopathology, immunohistology, chromatin immunoprecipitation (ChIP) sequencing, immunoblotting on 34 patients with radiographic sign of end-stage KOA.	Role of UTX, in concert with PRC2 core components, in controlling H3K27 tri-methylation and articular chondrocyte anabolism and OA development.
Lin X et al., 2020 [53]	DNA methylation	Methylation profiles on a public dataset from patients with hip/knee OA (470.870 CpG probes in 108 samples) vs. hip tissue—healthy controls.	16,816 differentially methylated CpGs, and nearly half (8111) of them were from enhancers, major DNA methylation changes in both types of OA in the enhancer regions. In KOA, 280 differentially methylated enhancer CpGs were identified.
Kreitmaier P et al., 2022 [59]	DNA methylation	Epigenome-wide association study of knee cartilage degeneration on 98 OA individuals.	Robustly replicating methylation markers, which reveal an etiologic mechanism linked to the migration of epithelial cells. Created a genome-wide methylation quantitative trait locus (mQTL) map of articular cartilage and synovium and identified 18 disease-grade-specific mQTLs.
Wu Z et al., 2022 [54]	DNA methylation	Analysis of the methylation pattern in knee and hip osteoarthritis on 16 OA hip samples, 19 control hip samples and 62 KOA samples.	12 methylation sites were identified; genes like MEIS1, GABRG3, RXRA and EN1.
Sarkar A et al., 2022 [55]	DNA methylation	EWAS, chromatine signature, STAT3 manipulation, unspecified sample size.	STAT3 regulates epigenetic status of cartilage cells, modulating DNA methilation in context-dependent manner.
Sorial AK et al., 2020 [56]	DNA methylation	Cartilage, fat pad, synovium and blood samples from TKA patients. A total of 240 samples from 202 patients.	Methylation correlates with PLEC expression. An allele of rs11780978 correlates with reduced PLEC expression and methyl at CpGs in cg19405177 and cg14598846. The rs11780978-PLEC eQTLs, mQTLs and meQTLs that are active in cartilage are also active in synovium (but with differences). PLEC mQTLs across all four tissues. Knocked-down plectin: increased expression of genes for immune response, decreased Wnt signaling (cartilage homeostasis).
Zhang Q et al., 2021 [57]	DNA methylation	PCR, Western blot and ELISA determination of epigenetic regulation determining TNF-a expression and its correlation to OA in 50 patients (37 OA + 13 controls).	Significant DNA hypo-methylation was observed in OA cartilage, DNA methylation of the TNF-a promoter suppresses mRNA and protein levels of TNF-a in OA, anacardic acid can inhibit the binding of HAT1 and CBP on the TNF-a promoter and reduce TNF-a secretion.
Zhou L et al., 2021 [28]	lncRNA	Analysis of the roles and associations of PCAT-1 and its target miR-27-3p in the pathogenesis OA on 30 articular cartilage samples (15 KOA + 15 healthy).	lncRNA PCAT-1-3 has a pro-apoptotic effect in KOA chondrocytes via regulation through sponging miR-27b-3p in KOA.
Fan H et al., 2021 [27]	lncRNA	Analysis on 40 samples (20 KOA + 20 healthy) of the expression of SNHG16 in OA and normal tissues. CHON-001 cells treated with interleukin (IL)-1β serve as an in vitro model of human OA.	lncRNA SNHG16 is up-regulated in KOA. lncRNA SNHG16 promotes the occurrence of KOA by sponging miR-373-3p and its target gene p21.
Van Hoolwerff M et al., 2020 [26]	lncRNA	Analysis of differential expression of lncRNAs in macroscopically lesioned and preserved articular cartilage on 98 samples (65 knees, 33 hips).	Antisense lncRNAs play an important role in regulating the pathophysiology of KOA. Antisense lncRNAs can exert their function in cis.
Li X et al., 2021 [30]	lncRNA	Analysis of existing bulk RNA sequencing (bulk RNA-seq) and single-cell sequencing (scRNA-seq) data for chondrocytes in OA knees, for identification of key transcription factors and lncRNAs on unspecified sample size.	271 key genes are suggested as involved in the pattern of progression of OA. For these genes, 14 transcription factors, among which TWIST2, MYBL2, RELA, JUN, KLF4 and PTTG1 are mentioned as the key TFs. A total of 8 lncRNAs among the 271 genes and the lncRNA regulation between CYTOR and NRP1 are shown to contribute to the pain and vascularization of cartilage in KOA.
Wang Y et al., 2021 [29]	lncRNA	Analysis of the role of lncRNA THUMPD3-AS1 in OA biology on 10 KOA.	lncRNA THUMPD3-AS1 is down-regulated in KOA cartilage tissues and IL-1b-stimulated chondrocytes. Over-expression of lncRNA THUMPD3-AS1 alleviates cell apoptosis and facilitates inflammatory responses. THUMPD3-AS1 increases the levels of inflammatory markers. NF-κB p65 and MAPk p38 are identified target proteins of phosphorylation.
Wang G et al., 2022 [31]	lncRNA, miRNA	Analysis of differential expression of lncRNAs and miRNAs in normal chondrocytes and in 10 SDF-1-induced models of chondrocyte degeneration.	186 lncRNAs have significant expression (88 up-regulated, 98 down-regulated). A total of 684 miRNAs have significant expression. Indication of 10 top core genes: CXCL10, ISG15, MYC, MX1, KOASL, IFIT1, RSAD2, MX2, IFI44L and BST2 associated with chondrocyte degeneration, by inducing expression of N-acetyl-b-d-glucosidase (NAG) and MMP.
Tavallaee G et al., 2022 [36]	miRNA	Human: synovial tissue samples from OA patients. Mouse: (1) knee joints collected DMM or sham surgery. For the injection experiments in mice, (2a) miRNA 27b-3p mimic within knees; knees retrieved 5 weeks after; (2b) miRNA 27b-3p inhibitor 1 and 3 weeks after DMM/sham surgery; knees retrieved 5 weeks after. Human: unspecified; mice: (1) 6–10 knees for each group (DMM and sham); (2a) 6 knees per group; and (2b) 10 cases vs. 9 controls.	Association between miRNA 27b-3p expression and OA severity. Transfection with the miR-27b-3p mimic induces pro-fibrotic responses, migration and expression of key ECM genes. RNA sequencing identification of a PPARG/ADAMTS8 signaling axis regulated by miR-27b-3p in OA FLS.
Liu Y et al., 2022 [45]	miRNA	Administration of exosomes enriched with miR-140.	Superiority of hUSC exosomes over-expressing miR-140-5p for treating OA.
Dou P et al., 2021 [46]	miRNA	Analysis of the relationship between DNMT3B and miR-29b and their implications in humans, 46 OA + 46 normal, and 48 DMM mice.	DNMT3B exerts an anti-apoptotic function by reducing RUNX2. DNMT3B inhibits the expression of miR-29b via DNA methylation. The inhibition of miR-29b increases PTHLH expression. PTHLH impedes the apoptosis of chondrocytes by elevating CDK4. Up-regulation of CDK4 by DNMT3B induces the ubiquitination of RUNX2 protein.
Liu P et al., 2022 [48]	circRNA	Real-time PCR identification of circular RNA (circRNA) expression profile in 5 OA and 5 controls.	hsa_circ_0072697 role in the pathogenesis of OA.
Wang Y et al., 2020 [49]	circRNA	qRT-PCR detection of circRNA in peripheral blood of OA patients (the Western Ontario and McMaster Index of stiffness and pain).	1627 differentially expressed circRNAs. hsa_circRNA_0020014 results to be the most significantly differentially expressed and may be identified as a new potential biomarker.
Wu Q et al., 2020 [50]	circRNA	RT-qPCR detection of circRNA HIPK3 on OA and its regulatory mechanism in human OA tissue (American College of Rheumatology) on 36 OA patients.	CircHIPK3 is highly expressed, and miR-124 is down-regulated in OA tissues, as they are negatively correlated. CircHIPK3 suppresses apoptosis of OA chondrocytes by the miR-124/SOX8 pathway.
Duran-Sotuela A et al., 2023 [62]	mtRNA	Identification of mitochondrial DNA genetic variants associated with the risk of rapid progression of KOA. Characterization of their functional significance with a cell model. OAI cohort (1095), hip (373) and knee (326).	mtDNA variant m.16519C is over-represented in rapid progressors. Cybrids with this variant show increased mtDNA copy number and decreased mitochondrial biosynthesis. Higher amount of ROS and less resistance to oxidative stress. Impairment of autophagic flux. Modulates the transcriptome of cybrids.
Park H et al., 2022 [63]	siRNA	Effects of p16INK4a si-RNA NPs (PLGA); unspecified sample size.	Expression of p16INK4a is increased in the synovium and articular cartilage from OA patients. “p16 si_NPs” reduced the levels of TNF-α, IL-1β and IL-6 in FLSs and MMP-13 in chondrocytes. p16 si_NP injection in the model alleviated pain-associated behavior and reduced cartilage damage.

## 4. Discussion

Deciphering epigenetic signatures offers a deeper understanding of the intricate mechanisms underlying diseases, with epigenetic features emerging as promising biomarkers for complex illnesses, a mean to shed light on the underlying pathogenesis and paving the way for identifying potential therapeutic targets [64,65,66,67,68,69,70].

Rather than permanent changes in DNA nucleotide sequence, musculoskeletal degenerative disease is often associated with epigenetic modifications. Many environmental factors, such as early trauma, socioeconomic and psychological factors, are relevant to its pathophysiology: such factors mediate the development of chronic pain, in part, through epigenetic mechanisms [71].

While the contribution of genetic and epigenetic factors to OA in general is well-recognized, their precise role in disease management remains an area of active research, as well as translation into clinical practice of the putative therapeutic targets that are being identified.

A total of 40 papers met the inclusion criteria of the present review, studying three principal epigenetic mechanisms involved in knee OA: DNA methylation, histone modification and RNA-mediated gene silencing.

DNA methylation represents the best-characterized epigenetic modification and involves the addition of methyl groups to cytosines, predominantly at the dinucleotide CpG. In turn, CpG methylation undergoes dynamic changes in correlation with environmental factors and plays an important role in gene expression regulation. More specifically, methylation dynamics in enhancer regions can up-regulate the expression of downstream genes. We found that seven studies explored some role of DNA methylation mechanisms on the development of OA in humans and two in the animal model.

Histone methylation and demethylation are epigenetic modifications that have the power to reduce or bolster gene expression, especially altering chromatin structure. Surprisingly, only one study in the animal model and one study in human samples presented data on histone modification; yet, both studies were able to suggest putative therapeutic targets with the potential to interfere with degeneration.

A non-coding RNA (ncRNA) is a functional RNA molecule that is not translated into a protein; its capacity to bind and silencing expression of coding RNA base its regulatory activity in cells; ncRNA includes miRNA, circRNA and lncRNA. Most studies in the period of interest focused on this mechanism of gene regulation: 28 (12 human and 16 animal models) out of 40. According to our review, the majority of studies explored the involvement of miRNAs in the occurrence and development of OA: 14 in the animal model and 4 in human tissues. The main reason for the interest is the putative role in targeting genes enriched in cytokine–cytokine receptor interactions, cell differentiation, the nuclear factor k-light-chain-enhancer of activated B cells (NF-kB) signaling pathway, the transforming growth factor (TGF-b) signaling pathway and the ion signaling pathway.

In this review, three studies on human tissues were found to be focused on circRNA differential expression profiles and one in the animal model. CircRNAs belong to the non-coding RNA family and, equivalently to lncRNAs and miRNAs, are widely involved in gene expression regulation and have been shown to play an important role in the development of different diseases, among which includes KOA.

Long non-coding RNAs are RNA transcripts with little or null protein-coding potential, deputed to regulate transcription and translation. Out of the articles considered, 6/15 found that long non-coding RNA was involved in OA.

Studies concerning samples derived from animal models were 19 in total. The majority (12/19) involved experiments aiming to unveil the mechanism of action (MoA) of an investigated regulatory molecule. Seven (7/19) were focused on putative therapeutic protocols involving treatments and their effect on cells or animal models suffering from iatrogenic post-traumatic knee OA.

Studies based on human specimens or human-derived cells were 21 in total. Most of them (15/21) identified mechanisms underlying the development and/or progression of knee OA. A total of 12 studies included pilot tests towards the identification of therapeutic targets, and 4 studies identified putative biomarkers of OA.

While analyzed studies provide valuable insights, it is critical to acknowledge their limitations to interpret their findings accurately.

Animal models offer practical and clinically relevant approaches to investigate both the natural progression and therapeutic response of KOA, where load and biomechanics are considered crucial contributing factors [72]. The animal model used in the studies were etiher mice or rats. The rodent knee models present challenges due to a significantly smaller size compared to humans, extremely thin cartilage layer, lacking distinct radial, transitional and superficial layers and lack of the human layer of calcified cartilage adjacent to the subchondral bone. Despite these limitations, rodent models have proved useful in elucidating the genetic and molecular pathogenesis; a direct comparison of epigenetic mechanisms between species is lacking, unlike what is available for gene studies. Due to species-specific protein variation, differences in the regulatory networks and mechanical conditions (biped vs. quadruped) may lead to transferability.

One of the most recurrent limitations we record in our review was the small sample size, as self-reported by authors in eight studies. Small sample size more likely leads to results due to random statistical variations rather than to any real effect. The small sample size was generally due to the need for multiple groups to account for tissue heterogeneity, to lack of funding, to the goal of the study (proof of concept) or to non-specified reasons.

The need for in vivo evidence to follow up experimental in vitro and bioinformatic studies was pointed out by all authors of such studies, and this was due to lack of funds in one report, to early-stage research in many cases and to non-specified reasons in all the others.

Discrepancies in sample size, type of samples or RNA-seq techniques prevented comparing results with previous studies, limiting most new data impact; only eight studies planned and then explicitly analyzed their consistency with previous studies.

Age, gender and BMI were not included in multivariate analyses, preventing the establishment of a definitive, stage-specific role of considered factors.

Including only known ncRNAs in KOA articular cartilage can potentially represent a systematic bias, as not all of the lncRNAs involved in OA are known a priori: in a matter of complex regulatory network, a critical mass of potential additional regulators needs to be investigated to properly model the biological significance in vivo, gaining predictive capability of general explanatory model validation.

Other limitations of most studies include heterogeneity, lack of replication and lack of generalizability. Heterogeneity refers to the fact that studies often use different methods and procedures, which can make it difficult to compare their data. Lack of replication refers to the fact that many results are not replicated by independent researchers, which makes it difficult to confirm their findings. Lack of generalizability refers to the fact that the results of a study may not apply to a wider population. Seven studies failed to analyze at all their own methodology and subsequently acknowledge any limits.

The main limitation of the present review is the high internal heterogeneity among the included studies in terms of study design, values of association and predictive capacity. This heterogeneity could have arisen for different etiologic features covered in the studies. Furthermore, substantial variability can be observed in molecular findings depending on the tissue type, DNA preparation and processing methods and the sensitivity of the employed techniques. These factors are compounded by the limited number of published studies and the lack of replicated studies to verify the most intriguing findings. The global heterogeneity in terms of the epigenetic factors investigated, type of observations (cells, animals, patients), study designs, data and analytical methods used prevents application of bias analytical tools, properly implemented when confronting randomized trial. The literature included in our review is not yet mature enough for such assessment; we recommend that such perspective be focused on the the design of future studies.

Currently, epigenetic testing is not part of standard clinical practice, but its potential to personalize treatment makes epigenetic profiling a promising future approach to degenerative musculoskeletal chronic pain management, replacing alternative or traditional categorization models [73]. To do so, much more evidence needs to emerge from coordinated and comprehensive studies with large samples, in vivo testing and multivariate analysis, possibly relying on machine learning tools to integrate dimensions.

In the foreseeable future, the integration of epigenetic biomarkers with clinical and radiographic parameters holds immense potential for the development of novel diagnostic classifications that incorporate prognostic capabilities for degeneration and chronic pain progression, alongside the emergence of novel therapeutic strategies harnessing the power of epigenetic modulators. To achieve this ambitious goal, the pursuit of prospective comparative studies employing well-structured architectures and cohorts becomes an indispensable endeavor.

## 5. Conclusions

Epigenetics presents a burgeoning frontier for unraveling the molecular determinants underlying OA, justifying further exploration in this realm. Molecular assays hold immense potential to revolutionize disease management strategies. This paradigm shift will hinge upon the identification of robust epigenetic biomarkers and elucidation of the underlying biological pathways. The literature overwhelmingly supports the strong association between epigenetic modulation and OA, with some of these epigenetic markers already proposed as therapeutic targets in other ailments. This offers prospects for their investigation as potential therapeutic targets in OA management. Some areas of epigenetic regulation in KOA appear to be under-represented in research, despite great interest in the preliminary results of the few related studies, i.e., histone activation and deactivation. To fully harness the transformative potential of epigenetics, additional data are indispensable, alongside rigorous studies meticulously examining the tissues implicated in the pathology of low back pain. These studies should adhere to prospective designs, incorporate homogeneous cohorts of patients with consistent demographic characteristics, employ standardized sample processing protocols and employ rigorous technical evaluations in integration with previous published research.

## Data Availability

All data supporting reported results is included in the article itself; further details on the methodology are available from corresponding author upon reasonable request.
